# Maternal transfer of the most potent peanut allergen Ara h 2 into human breast milk in a German cohort

**DOI:** 10.1186/2045-7022-5-S3-P24

**Published:** 2015-03-30

**Authors:** Frauke Schocker, Joseph Baumert, Skadi Kull, Arnd Petersen, Uta Jappe

**Affiliations:** 1 Division of Clinical and Molecular Allergology, Research Center Borstel, Member of the German Centrer of Lung Research, Borstel, Germany; 2 Food Allergy Research and Resource Program, University of Nebraska, Lincoln, NE, USA

## Background

Peanut allergy is known to be one of the most severe food allergies. The aim of our breast milk study was to investigate the transfer of the most potent peanut allergen Ara h 2 into human breast milk in a German cohort. Therefore, the time courses of appearance after ingestion, the potential risk of sensitization to peanuts via breast milk in Germany and, if possible, the way Ara h 2 will be processed *in vivo* after secretion into breast milk was studied.

## Methods

Of 32 lacating, non-peanut allergic women, breast milk samples were collected at different time points after ingestion of 100 g dry roasted peanuts (approved by the local ethics committee). Breast milk samples were analysed for peanut protein in SDS-PAGE, Western blot and ELISA (Neogen Veratox ELISA^®^ and an ELISA against digestion resistant Ara h 2 (DRP-Ara h 2)). Natural Ara h 2 was digested by Enzynorm f^®^ and Kreon^®^ to mimic the effect of the combined gastric and duodenal digestion *in vivo* and, subsequently, analysed by N-terminal sequencing and MALDI TOF MS.

## Results

Ara h 2 was undetectable using Western blot. Performing the Neogen Veratox ELISA^®^ against crude peanut extract peanut proteins still remained undetectable. However, Ara h 2 was identified by an ELISA against DRP-Ara h 2 in 8/32 women (25 %) at different concentrations and time points of appearance. To assess the way Ara h 2 is processed *in vivo*, natural Ara h 2 was digested into several digestion resistant immunoreactive peptides <15 kDa after treatment with Enzynorm f^®^ and Kreon^®^, and a 12 kDa fragment was identified by N-terminal sequencing and mass spectrometry corresponding to the middle part of Ara h 2.

## Conclusions

After maternal ingestion Ara h 2 is secreted into breast milk in our German cohort in 25 % of the volunteers, individually either rapidly (after 1h, 2h or 4 h) or delayed (after 8h or 12h) and in different concentrations. To study Ara h 2 or Ara h 2 peptides that survive digestion and pass into human breast milk antibodies against the 12 kDa fragment are now raised for enrichment strategies to characterize these sensitizing or tolerogenic peanut structures in our breast milk samples.

